# Prevalence of Depression among Geriatric Population in a Rural Municipality of Nepal: A Descriptive Cross-sectional Study

**DOI:** 10.31729/jnma.8781

**Published:** 2024-10-31

**Authors:** Maheshor Kaphle, Rajesh Karki, Anjana Thapa, Ramesh Bhatt

**Affiliations:** 1 Department of Public Health, Peoples Dental College and Hospital, Naya Bazar, Kathmandu, Nepal; 2 Central Department of Public Health, Institute of Medicine, Tribhuvan University, Kathmandu, Nepal; 3 Ask Foundation, Kathmandu, Nepal; 4 Department of Public Health, Yeti Health Science Academy, Kathmandu, Nepal

**Keywords:** *elderly*, *geriatric depression*, *prevalence*, *rural population*

## Abstract

**Introduction:**

Depression is a major public health concern among the elderly, affecting their quality of life and overall well-being. Despite the increasing elderly population in Nepal, data on the prevalence of depression in rural areas is limited. Therefore, this study aims to assess the prevalence of depression among the geriatric population.

**Methods:**

A cross-sectional study was conducted among individuals aged 60 years and above in three randomly selected wards of Shivapuri Rural Municipality. Respondents were selected using consecutive sampling. The Nepali version of the Geriatric Depression Scale (GDS-15) was administered through face-to-face interviews. Ethical approval was obtained from the Institutional Review Committee (Reference number 080/81-437). Descriptive analysis was conducted for categorical variables using the Statistical Package for the Social Sciences (SPSS).

**Results:**

The overall prevalence of geriatric depression was 115 (28.75%; 95% CI: 24.36%-33.46%), with a mean depression score of 3.54±3.17. Among those with depression, majority reported mild depression 79 (68.70%) reported mild depression, 79 (31.22%) were male, 88 (30.66%) were under 75 years of age, 46 (35.66%) self-rated their health as not good, 16 (32.66%) had COPD and 33 (30%) were hypertensive elderly.

**Conclusions:**

About one third of the elderly reported of having depression and more than 50% had mild depression. The depresson was more in age group less than 75 years, male, married and in nuclear family.

## INTRODUCTION

The rapidly growing geriatric population in developing nations poses a severe challenge to existing mental health treatments.^1^ Depression impacts individuals in every community worldwide and plays a major role in the global burden of disease.^2^ Depression was found to be the third most common cause of years lost to disability.^3^ Undiagnosed depression with other comorbidities such as physical impairment, anxiety, and other general medical problems.^4^ As per WHO, mental illness affects 14% of persons 60 years of age and older.^5^ Based on the findings of earlier research, more than half (53.1%) of elderly people in community settings had depression.^6^ Most of these studies are done in aged care homes with very few in community settings. This study aims to assess the prevalence of depression among the geriatric population of Shivapuri Rural Municipality, Nuwakot, Nepal.

## METHODS

A cross-sectional survey was conducted among the geriatric population of Shivapuri Rural Municipality, Nuwakot District, from April to June 2024. Ethical approval was obtained from the Institutional Review Committee (IRC) of Yeti Health Science Academy (Ref. no: 080/81-437). Written informed consent was obtained from all respondents before data collection. Anonymity and confidentiality were maintained, and participation was voluntary. Participants aged 60 and above who were present at home during the survey and voluntarily agreed to participate were included. Those diagnosed with mental illness by a physician or who refused consent were excluded. The sample size was calculated using the formula:


$$\begin{array}{ll} \text{n} & ={\text{Z}}^{2}\times \frac{\text{p}\times \text{(1}-\text{p)}}{{\text{e}}^{\text{2}}}\\ & =1.96^{2}\times \frac{\text{0.6804}(\text{1}-\text{0.6804})}{0.05^{2}}\\ & =344.01 \end{array}$$

where,

Z = 1.96 at 95% Confidence Interval (CI)
P = 68.04%^7^ (prevalence of depression among the geriatric population from a previous study)
q = 1-p
e = margin of error, 5%

The calculated sample size was 334. Adding a 20% non-response rate,^6^ which gives 400. We included 397 respondents because three respondents did not provide complete information. Shivapuri Rural Municipality has 8 wards. Three wards (5, 6, and 8) were selected randomly using a lottery method. Consecutive sampling was employed to recruit participants from households with eligible individuals. If multiple eligible participants were present in a household, all were included.

Socio-demographic variables (age, sex, ethnicity, religion, education, income source, and socio-economic status) and health-related variables (self-rated health status, hypertension, COPD, and smoking behaviors) were collected. Economic status was measured using wealth index.^8,9^ Hypertension was assessed by those who are taking antihypertensive medicine, taking blood pressure, and cross-verified with medical records. Respondents were classified as having hypertension based on their medical history, specifically, if they were taking antihypertensive medication at the time of the survey or as indicated in their medical records.^10^ For those not on medication, blood pressure was measured by trained health personnel. Medical records were used to verify the presence of COPD, and smoking status was self-reported.

The Nepali version of the Geriatric Depression Scale (GDS-15) was used to measure depression.^11^ Data were collected via face-to-face interviews. Before the main survey, the GDS-15 was pretested among 30 eligible respondents in Tokha Municipality, Ward No. 8. Based on the pretest findings, minor modifications were made to the sociodemographic questions and those related to perceived health conditions. The reliability of the GDS-15 for our study was confirmed with a Cronbach's alpha coefficient of 0.796. The GDS-15 items were scored as follows: Five items (1, 5, 7, 11, and 13) indicated depression when answered "no." Ten items (2, 3, 4, 6, 8, 9, 10, 12, 14, and 15) indicated depression when answered "yes." Total scores ranged from 0 to 15, with the following classifications: 0-4: No depression, 5-8: Mild depression,9-11: Moderate depression, and ≥12: Severe depression.^12^ Self-rated health was measured by a single-item question, "In general would you say your health is poor, fair, good, or excellent?" Poor and fair were recoded as "Bad" and good and excellent were recoded as "Good".^13^

The wealth index was measured by the International Wealth Index (IWI)^8^ in the poorest, poor, middle class, rich, and richer and dichotomized to up to middle class and rich.

Collected data were entered into Epi-Data version 3.1 and analyzed using SPSS version 25. Variables were dichotomized for descriptive analysis. Descriptive statistics (frequency, percentage, median, standard deviation) summarized socio-demographic characteristics and the prevalence of depression. Crude prevalence with a 95% confidence interval was calculated.

## RESULTS

The study included 400 geriatric patients however as three respondents did not provide complete information the total sample for this study was 397. The median age was 70 (IQR: 64-75) years, male were 253 (63.73%) and Hindu were 296 (74.66%). There were 276 (69.52%) elderly who could not read or write (Table 1).

**Table 1:** Socio-demographic characteristic of the sample geriatric population in Shivapuri rural municipality (n=397).

Variables	n (%)
Age	Median (Q1-Q3): 70.00 (64-75)
Sex	
Male	253 (63.73)
Female	144 (36.27)
**Religion**	
Hindu	296 (74.56)
Buddhist	94 (23.68)
Christian	6 (1.51)
Others, Islam	1 (0.25)
**Education**	
Cannot read and write	276 (69.52)
Can only read and write (no formal education)	69 (17.38)
Basic Level Education	31 (7.81)
Secondary-level education or above	21 (5.29)
**Main source of Income**	
Agriculture	345 (86.90)
Job	24 (6.05)
Business	14 (3.53)
Labor	7 (1.76)
From remittance of migrant workers	7 (1.76)
**Wealth index**	
Up to middle class	236 (59.45)
Rich	161 (40.55)
**Marital status**	
Married	305 (76.83)
Widow	85 (21.40)
Unmarried	4 (1.01)
Separated	3 (0.76)

Of the total respondents (n=397), the prevalence of geriatric depression was 115 (28.75%; 95% CI: 24.36%-33.46%), with a mean depression score of 3.54±3.17 (range: 0-15). (Table 2).

**Table 2:** Prevalence and severity levels of geriatric depression among respondents and self-perceived health status (n=397).

Variables	n (%)
**Prevalence of depression**	
Yes	115 (28.75)
No	282 (71.04)
**Depression level (n=115)**	
Mild	79 (68.70)
Moderate	26 (22.60)
Severe	10 (8.70)
**Depression Score**	
Mean (SD)	3.54±3.17

Regarding co-occurring health conditions, 131 (32.99%) reported hypertension, 66 (16.63%) reported COPD and 159 (40.05%) reported currently smoking tobacco products (Table 3).

**Table 3:** Self-reported health among the geriatric population (n=397).

Disease/Health condition	n (%)
**Hypertension**	
Yes	131(32.99)
No	266(67.01)
**COPD**	
Yes	66 (16.63)
No	331 (83.37)
**Smoking**	
Yes	159 (40.05)
No	238(59.95)
**Self-rated health status**	
Good	268 (67.50)
Bad	129 (32.49)

Geriatric depression was observed in 88 (30.66%) of geriatric population less than 75 years of age (Table 4).

**Table 4:** Cross-tabulation of selected demographic variables with geriatric depression (n=397).

Variables	Geriatric depression	
Age	Yes	No
	n(%)	n(%)
<75 Years	88 (30.66)	200 (69.44)
≥75 Years	27 (24.88)	82 (75.22)
**Sex**		
Male	79 (31.22)	174 (68.77)
Female	36 (25.00)	108 (75.00)
**Religion**		
Hindu	86 (29.06)	210 (70.94)
Buddhist	25 (26.59)	69 (73.41)
**Family type**		
Nuclear	58 (30.36)	133 (69.63)
Joint	48 (25.94)	137 (74.06)
Extended	9 (42.86)	12 (57.14)
**Marital status**		
Married	94 (30.82)	211 (69.18)
Unmarried/widowed/separated	21 (22.82)	71 (77.17)
**Education**		
Cannot read and write	78 (28.27)	198 (71.73)
Can only read and write (no formal education)	20 (28.99)	49 (71.01)
Basic Level Education	7 (22.58)	24 (77.41)
Secondary-level education or above	10 (47.62)	11 (52.38)
**Wealth index**		
Up to middle class	78 (33.06)	158 (66.94)
Rich	37 (22.99)	124 (77.01)

Amongst the respondents who self-reported poor health status, 46 (35.66%) had depression. Among those with hypertension, 33 (30.00%) reported depression. Similarly, 16 (32.66%) patient with chronic obstructive pulmonary disease also reported depression (Table 5).

**Table 5:** Cross-tabulation of self-perceived health status with geriatric depression (n=397).

Variables	Geriatric	Depression
	Yes, n (%)	No, n (%)
Self-rated health Status		
Good	69 (25.74)	199 (74.25)
Not Good	46 (35.66)	83 (64.34)
**Hypertension**		
Yes	33 (30.00)	77 (70.00)
No	82 (28.58)	205 (71.42)
**Reported COPD**		
Yes	16 (32.66)	33 (67.34)
No	99 (28.45)	249 (71.55)
**Smoking tobacco product**		
Yes	63 (37.06)	107 (62.94)
No	52 (22.91)	175 (77.09)

## DISCUSSION

Among 397 elderly people, nearly one-third (28.96%) were depressed. A similar study conducted among the elderly population among the Rai ethnic group of Nepal found that 29.7% of older adults were depressed.^14^ Another study conducted in a rural municipality of Rauswa district of Nepal found a consistent result.^15^ The prevalence of geriatric depression varies in different studies in Nepal and the world. A study in a community setting from South Korea found that three among five Korean elderly had depression measured by the same scale. The mean score for depression was 6.21 (±3.83) which was almost double higher than our study.^16^ Due to differences in cultural norms and societal attitudes between Korea and Nepal towards parent care, they may be different. In Nepal, Joint family practice is common and constitutes higher in this study which helps to provide support and care to the elderly at home. Another study conducted in Bangladesh found that more than half 54.6% of the elderly population from urban and rural areas had depression.^17^ This discrepancy could be linked to differences in socioeconomic factors, access to healthcare, and poverty levels among the elderly. Another study conducted in rural Odisha; India found that 44.4% of the elderly had geriatric depression^18^ which was also higher than our study results. This difference might be due to regional variations in healthcare access, physical health status, and social support. However, another study conducted in Jammu & Kashmir of north India in a community setting found a lower result than the Odisha study. It was almost 39% of the elderly had geriatric depression.^19^ These findings indicate regional disparities in depression prevalence, possibly influenced by socioeconomic and cultural factors. A study to find out the prevalence and factors associated with depression among the hill tribe elderly population in Thailand found that nearly one-third of them had depression^20^ which was consistent with our study. The elderly in Thailand and Nepal share similar challenges, including social isolation, economic hardship, and limited access to healthcare, which could explain the comparable prevalence rates. A systematic review and meta-analysis done in India found almost one-third of the elderly had geriatric depression,^21^ and a global systematic review reported a similar finding,^22^ further supporting the consistency of our results with larger-scale data.

This study found the prevalence of geriatric depression was higher in men. About one-third of men and one-fourth of females exhibit symptoms of depression. This difference is not noteworthy but may be due to several reasons. One of the important causes may be the marital status including the death of a spouse and divorce. Nearly one-third of the elderly were separated, widowed, or unmarried and the number of men was high. This fact was also supported by a study conducted in India.^18^ but another review study reported that 81% of the 85 studies found women were more likely to have depression.^23^ This suggests that gender differences in depression may vary based on cultural or regional contexts. Educational status and socioeconomic status also play a role in geriatric depression. The study found that low socioeconomic status and education level experienced more depressive symptoms.^24^ There was no significant difference in the percentage of literate and illiterate elderly having depression. Still, in the wealth index, individuals who fall up to the middle class exhibit a higher rate of depression which is consistent with other studies.^18,25^ This could be linked to financial insecurity and limited access to resources among the lower socioeconomic groups.

The health status of an individual may have a crucial role in the appearance of geriatric depression among the elderly. We assessed the perceived health status of the elderly. They rate their health status as either good or not good. We found that those who rated their health status as "not good" had a higher rate of depression which is consistent with other studies.^16,^ ^26^ This study revealed that elderly people who had hypertension, and COPD, and current smokers have higher rates of geriatric depression. The findings of this study also support other studies from Thailand,^19^ India,^18^ Bangladesh,^26^ and Nepal.^6^

This study tried to identify the situation of depression among the geriatric population in the community setting. So, the findings from the hospital-based study may differ from this study. Due to the limited sample size, we cannot predict the actual causes of depression among the elderly population. The nationwide geriatric survey may be needed to estimate the actual prevalence of depression in Nepal.

## CONCLUSIONS

The findings revealed a concerningly high prevalence of depression (28.9%), with a higher rate among those under 75 years old and male. The elderly depression was seen more in male gender, lower education levels and poor self-reported health, and chronic health conditions like hypertension and COPD. A higher prevalence of depression was observed in nonsmokers compared to smokers.
